# Exploration of underlying patterns among conflict, socioeconomic and political factors

**DOI:** 10.1371/journal.pone.0304580

**Published:** 2024-05-31

**Authors:** Kathleen Vazquez, Jeffrey C. Johnson, David Griffith, Rachata Muneepeerakul

**Affiliations:** 1 Agricultural and Biological Engineering Department, University of Florida, Gainesville, Florida, United States of America; 2 Department of Anthropology, University of Florida, Gainesville, Florida, United States of America; 3 Department of Coastal Sciences, East Carolina University, Greenville, North Carolina, United States of America; St John’s University, UNITED STATES

## Abstract

The emergence of conflict is a complex issue with numerous drivers and interactions playing a role. Exploratory dimension-reduction techniques can reveal patterns of association in such complex data. In this study, an existing dataset was reanalyzed using factor analysis for mixed data to visualize the data in two-dimensional space to explore the conditions associated with high levels of conflict. The first dimension was strongly associated with resilience index, control of corruption, income, income inequality, and regime type, while the second dimension was strongly associated with oil production, regime type, conflict level, political terror level, and water stress. Hierarchical clustering from principal components was used to group the observations into five clusters. Country trajectories through the two-dimensional space provided examples of how movement in the first two dimensions reflected changes in conflict, political terror, regime type, and resilience index. These trajectories correspond to the evolution of themes in research on conflict, particularly in terms of considering the importance of climate or environmental variables in stimulating or sustaining conflict. Understanding conditions associated with high conflict can be helpful in guiding the development of future models for prediction and risk assessment.

## Introduction

The emergence of conflict is a complex issue with numerous drivers and interactions playing a role. Data-driven models to predict national and subnational conflict are difficult to construct, with different approaches reaching different conclusions [1–3], but remain a sought-after tool in risk management. However, potential drivers of conflict are so numerous and complex that predictive success is low and causality is difficult to determine [1]. For example, previously developed models have incorporated a wide range of drivers, from ethnic relationships and geographic location to gender relations and perceived opportunities [4–8]. These drivers frequently do not act alone: complex interactions are often determining factors in whether or not violent conflict actually occurs [1, 6]. Appropriate analysis methods are needed to extract the patterns of these drivers and their interactions. Once analyzed, these patterns are useful for development of models and theories.

Van Holt *et al*. [9], in a critical path analysis of the conflict literature, found that datasets played an important role in advancing both modeling and theory in conflict research. In the multiple databases employed across studies, they found that there were a vast number of variables incorporated in modeling efforts to account for conflicts in various settings and contexts, yielding a complex array of possible theoretically meaningful explanatory variables and ultimately theoretical explanations. Exploratory statistical methods, such as those conducted in this paper, have a long history of being used to generate explanatory theories and have been referred to as an inductive method of theory generation and as a latent variable method [10]. Thus, such exploratory methods can serve as powerful tools for theory development and modeling by revealing hidden patterns, reducing complexity, increasing the chances for the development of more valid research hypotheses, and guiding future research efforts.

Previous studies, such as the Global Conflict Risk Index report for the European Commission [4], have relied on linear regression models to identify key drivers and generate predictions. This method neglects potential nonlinear relationships and threshold behavior, as well as the mixed nature of the different datasets. Categorical variables in the study were converted to numerical scales that were input into the linear regression approach. For truly categorical variables, such as regime type, values do not fall on an ordered numerical scale—‘Partial democracy’ is qualitatively different from ‘Partial autocracy’, rather than being one unit higher on an ordered scale. Treating these variables as truly categorical and applying the analysis techniques specifically designed for such variables would allow a more nuanced and accurate understanding of their relationship with conflict.

Revisiting the dataset used in the Global Conflict Risk Index report with a more compatible exploratory dimension-reduction techniques can reveal patterns of association not seen previously. One such method, principal components analysis, has been used to evaluate the ability of a complex dataset to classify nation-states with respect to violent conflict [11]. It has also shown potential to improve model forecasts of conflict when used with clustering algorithms [12]. Factor analysis for mixed data (FAMD), which uses principal components analysis as a foundation, is an exploratory dimension reduction technique that can be applied to more complex datasets containing both categorical and numerical data. This method and others like it are not for assigning causality, only for exploring connections and associations. The result of this method is new coordinates for the observations in terms of the extracted dimensions, so the data can be visualized in a lower dimensional space. This method is more compatible with the kind of mixed data relevant to understanding violent conflict, but has not yet been applied to socioeconomic and political data for better understanding of conflict patterns.

In this study, a portion of the Global Conflict Risk Index report dataset was reanalyzed using FAMD along with hierarchical clustering from principal components to answer the following research questions: What are the conditions associated with high levels of conflict. Is there any typology to illustrate these associations? Can we use this typology and visualization to understand the changes in a given country through time?

## Methods

In this study, a dataset of 14 categorical and numerical variables was analyzed using FAMD to explore the underlying patterns in the data through dimension reduction. Projecting the 14-dimensional data onto the first two extracted dimensions provides useful visualizations that help extract its underlying patterns. Hierarchical clustering was then performed on these FAMD-extracted dimensions, resulting in a typology of country-year characteristics. These clusters visualized in the reduced dimension space deepen our understanding of the conditions associated with high levels of conflict. Country trajectories through the reduced dimension space are then examined through examples, giving additional contexts to understand the changes in a given country through time.

### Factor Analysis of Mixed Data (FAMD)

Factor analysis of mixed data is a dimension reduction technique used to visualize patterns in large, highly-correlated datasets in a low-dimensional space [13]. The goal is to project the data onto orthogonal dimensions such that the captured data variability is maximized. FAMD effectively combines principal component analysis for numerical data and multiple correspondence analysis for categorical data, which allowed the dataset to be evaluated consistently as a whole. To include both types of data, the correlation coefficient is used to quantify the relationship with quantitative variables, while the squared correlation ratio is used to quantify the relationship with categorical variables [13]. Based on these two metrics, FAMD extracts dimensions that best illustrate the structure of the data as a whole. This method is an exploratory technique to provide insight into underlying data structure, rather than a tool to model or predict conflict outcomes. The FAMD function from the R package *Factominer* was used for this analysis [14].

### Hierarchical Clustering from Principal Components (HCPC)

Hierarchical clustering from principal components was used to identify clusters of observations in the dataset. HCPC used the dimension results from FAMD for dimensions with eigenvalues greater than one to produce the initial hierarchical clusters. Clusters were then consolidated using a k-means algorithm: Initial clusters were assigned and the center of gravity of each was calculated. Then, observations were assigned to the cluster whose center of gravity was closest. Cluster centers of gravity were then recalculated and observations were reassigned. This was repeated iteratively until clusters stabilized. The HCPC function from the R package *Factominer* was used for this analysis [14].

### Data

Data used in the Global Conflict Risk Assessment Report [4] were reviewed and variables with sufficient data coverage for the years 2000–2015 were consolidated for use in this analysis. A total of 10 quantitative and 4 categorical variables covering 156 countries over 16 years were used. Variable names, definitions, and summary statistics are summarized in Tables 1 and 2. Data was consolidated into a single dataset with each observation representing a set of country-year characteristics (the characteristics of a given country in a given year).

**Table 1:** Ten numerical variables included in the analysis.

Variable	Definition	# of Obs	Mean	St. Dev.	Min	Max
**ND-GAIN**	Climate hazard resilience index based on vulnerability and readiness	2112	48.7	11.37	26.99	77.83
**CCest**	Control of Corruption index measuring perception that public power is being used for private gain	1980	-0.04	1.05	-1.87	2.47
**Ginidisp**	Disposable income inequality index	1851	39.3	8.4	22.4	66.2
**Oilprod**	Oil production as percent of merchandise exports	1821	16.9	27.1	0	99.9
**UnEmp**	Unemployment rate	2112	7.55	5.53	0.17	35.27
**Population**	Total population size (thousands)	2112	44789	159002	413	1406848
**InfMort**	Mortality rate under 5 (per 1,000 live births)	2112	45.6	47.8	2.2	234
**Undernourish-ment**	Probability that a random citizen is undernourished	1934	12.7	12.7	2	71.5
**Food variability**	Food price variability	2075	13	13.7	0.1	107.7
**Waterstress**	Baseline water stress	2064	2.0	1.6	0.0	5.0

**Table 2:** Four categorical variables included in the analysis.

Variable	Definition	# of Obs	Categories	Freq.
**RegType**	Regime Type	2102	Full Autocracy	298
			Partial Autocracy	255
			Partial Democracy w/ Factionalism	292
			Partial Democracy	668
			Full Democracy	522
			Transitional	15
			Interregnum	25
			Interruption	27
**PTSC**	Level of repression/political terror	2105	1, Torture and political imprisonment/murder is extremely rare	341
			2, Limited imprisonment for nonviolent political activity, political murder is rare	570
			3, Extensive political imprisonment, unlimited detention for political views is accepted	721
			4, Murders/disappearances/torture are common, but terror primarily affects those involved in politics	346
			5, Terror of level 4 extended to whole population	127
**Conflict**	Battle related deaths per year, best estimate	2112	<10	1616
			10–100	168
			100–1,000	190
			1,000–10,000	131
			>10,000	7
**GDPpC**	GDP per capita (Constant 2017 international $)	2081	Low income, <1200	113
			Middle income, >1200 & <13000	1065
			High income, >13000	903

Among the included variables was the ND-GAIN Resilience index: it is comprised of two values, a vulnerability score and a readiness score [15]. The vulnerability score indicates a country’s overall vulnerability to climate hazards. The readiness score indicates a country’s ability to adapt and respond to climate hazards through economic, governance, and social structures. Conflict data was taken from the Uppsala Conflict Data Program [16]. Conflict numbers represent a best estimate of battle-related deaths per year. More information on this data can be found in Ref. [16].

Regime type was constructed from two variables describing the competitiveness of political participation and the method of executive recruitment (transfer of executive power). More details can be found in Ref. [17]. Categorical variables for conflict and gross domestic product (GDP) per capita were transformed from numerical to categorical for this analysis. This transformation has two benefits: allowing for nonlinear associations to be represented in dimension reduction, and curbing the visual influence of strong outliers in the data. Three categories of GDP per capita were defined based on World Bank definitions of low, middle, and high income countries [18]. Six categories of conflict were defined (Table 2).

Factor analysis of mixed data requires a complete dataset. While the dataset assembled here was relatively complete (<4% missing), the missing values needed to be imputed. The missFAMD function in the R package *missMDA* was used for this [19]. This function uses a regularized iterative FAMD algorithm. Missing values are first assigned a value: the mean of numerical variables or the proportions belonging to each category for categorical variables. Then FAMD is run. Based on the assumption that the first few extracted dimensions contain both information and noise, and the last dimensions contain only noise, the variance of the noise is estimated and removed from the first dimensions before they are used to predict the missing values; this is done to reduce overfitting issues. Then FAMD is run again with the new values. This process is repeated iteratively until convergence. Because FAMD is used to estimate missing values, the percentage of variability explained by the extracted dimensions will be slightly inflated, but with such a small percentage of missing values, this inflation is minimal. More information on this R package and the imputation technique used can be found in Refs. [19, 20].

## Results and discussion

### FAMD extracted dimensions

**Fig 1:**
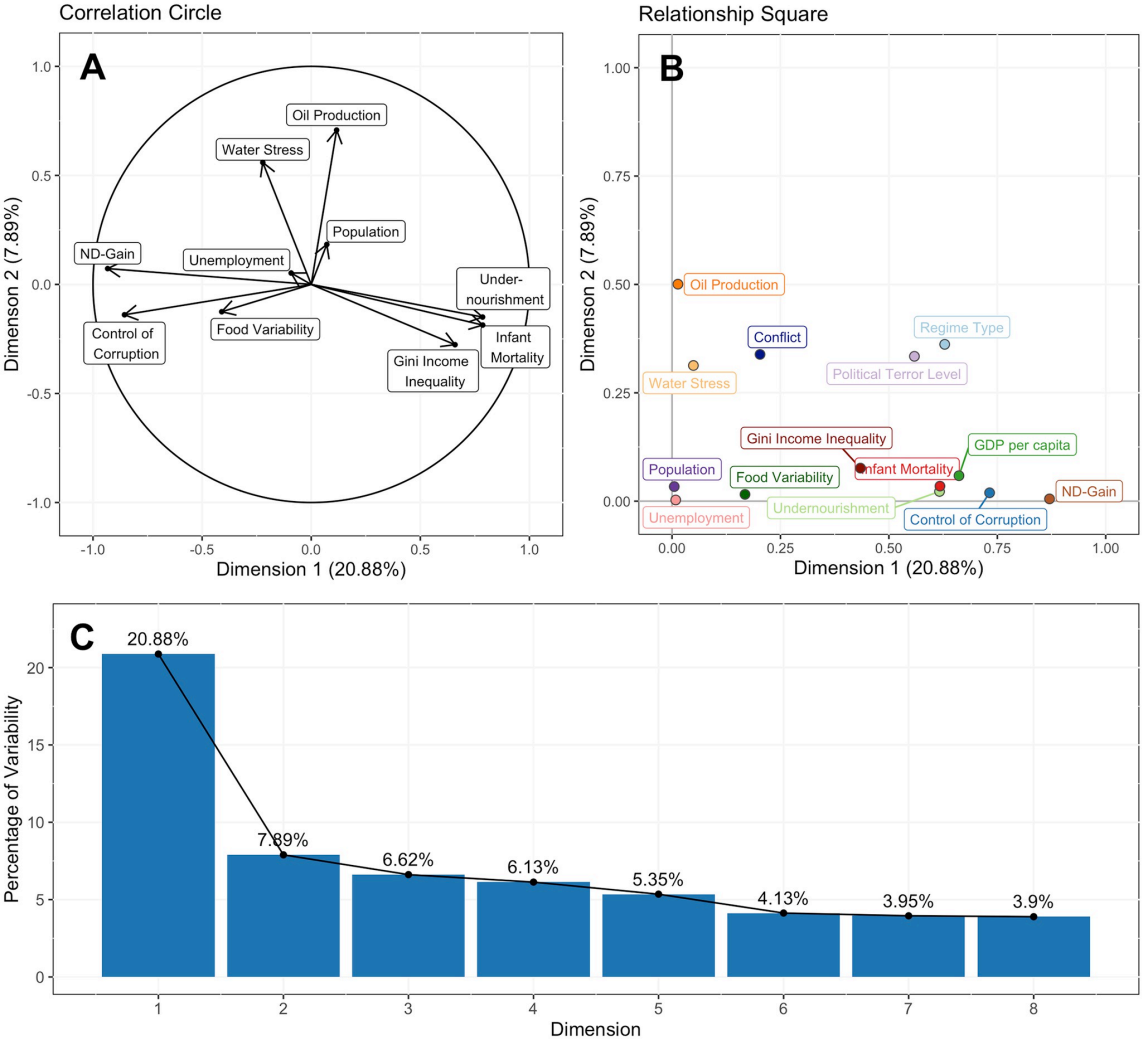
Factor analysis for mixed data results. **A)** The strength (length of arrow) and direction (direction of arrow) of correlation of each numerical variable with respect to the first and second dimensions. **B)** All variables, both numerical and categorical, and their relationship strength to the first two dimensions. **C)** The first eight dimensions extracted using FAMD and their respective explanation of data variability.

Applying FAMD to the dataset resulted in eight dimensions with eigenvalues greater than one, explaining a total of 58.85% of the variability of the dataset (Fig 1C). This cutoff of eigenvalues greater than one is known as the Kaiser criterion, and implies that an extracted dimension explains more of the data variability than any one of the original variables [21]. While more robust methods of selecting the number of dimensions for analysis exist, this is sufficient for exploratory analysis of highly interconnected data. We will focus on the first two dimensions, explaining 20.88% and 7.89% of data variability, respectively. These somewhat low values are not uncommon for complex and highly interconnected datasets. From Fig 1A, resilience index (ND-GAIN, Table 1) and control of corruption have a strong negative correlation with the first dimension, while income inequality, undernourishment and infant mortality have a strong positive correlation. The second dimension is most strongly correlated with oil production level and water stress. Regime type and political terror level are associated with both dimensions (Fig 1B). Conflict level is more strongly related to the second dimension.

### Typology of country-year characteristics

**Fig 2:**
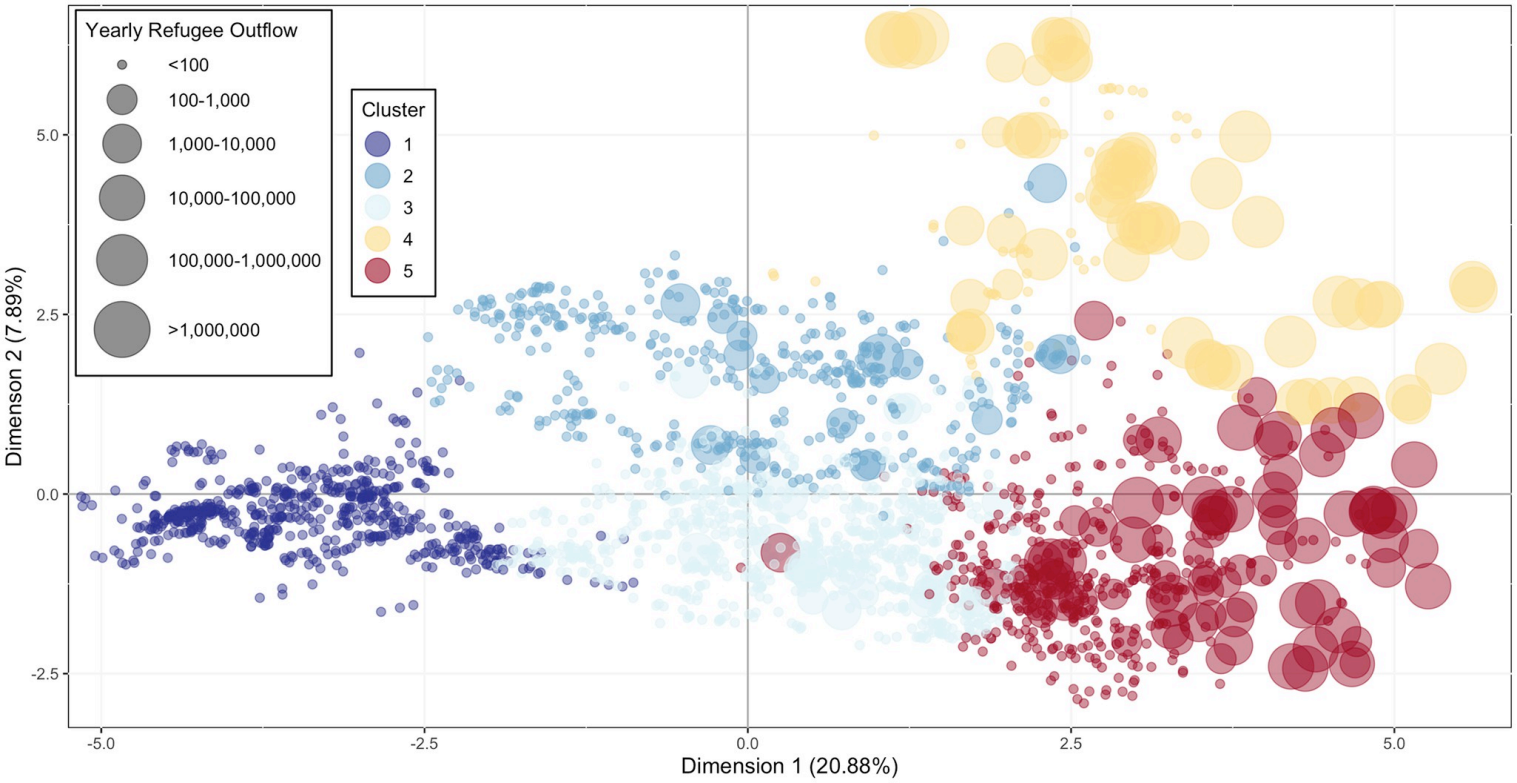
Clusters resulting from hierarchical clustering analysis. Each point in the map represents one country-year observation (one country in one year), which cluster it belongs to, and where it falls with respect to the first two dimensions. The data points are color-coded according to the clusters to which they belong. The size of a data points reflects the level of outgoing refugee flows from the corresponding country in the corresponding year. Note that the refugee data is used here for visualization purposes only and not part of the FAMD analysis.

Setting the clustering algorithm to extract five clusters resulted in sensible typology. The results are represented as the so-called graph of individuals [13] (country-year observations in this case), hereinafter referred to as the “map,” in the plane of the first two dimensions (Fig 2). Cluster membership of each country in each year is presented in the S1 Table. The characteristics of the five clusters are described below:

1. *High income democracies (n = 599 country-year observations, 42 distinct countries represented)*: Cluster 1 is most strongly associated with a regime type of ‘Full Democracy’, high income, high control of corruption, and high resilience index. The majority of European countries included in this analysis are represented in this cluster, along with the United States, Canada, Australia, Costa Rica, Chile, and several others. This cluster sees very little conflict and is associated with a healthy population (low infant mortality and undernourishment).
2. *Autocracies with high oil production (n = 459 country-year observations, 22 distinct countries represented)*: Cluster 2 is associated with a regime type of ‘Full Autocracy’, high oil production, and high water stress. Represented in this cluster is the majority of Western and Southeast Asia, most of Northern Africa, along with China, India, and Mexico. This cluster is made up of middle and high income countries that see some conflict and have a higher political terror level (PTSC of 3–4).
3. *Middle income partial democracies (n = 844 country-year observations, 62 distinct countries represented)*: Cluster 3 is the largest cluster and is most strongly associated with a regime type of ‘Partial Democracy’ and middle income. This cluster has higher income inequality and unemployment and a moderate political terror level (PTSC of 2–3), but sees very little conflict. There are 73 countries (across different years) represented in this cluster, covering a wide geographic range.
4. *High political terror, high conflict observations (n = 115 country-year observations, 13 distinct countries represented)*: Cluster 4 is the smallest cluster and is associated with the highest political terror level and highest conflict. All regime types are represented in this cluster with the exception of ‘Full Democracy’, and it contains the highest proportion of disrupted regime types (‘Interruption’, ‘Interregnum’, ‘Transitional’). This cluster is majority middle income, and it is associated with higher oil production. There are 25 countries across different years represented in this cluster, including Afghanistan, Colombia, Iraq, Pakistan, Rwanda, Sudan, Syria, and Ukraine.
5. *High undernourishment and infant mortality observations (n = 479 country-year observations, 36 distinct countries represented)*: Cluster 5 is most strongly associated with low indices of health—high infant mortality and high rate of undernourishment. It is also associated with low income and a regime type of ‘Partial Autocracy’, though several regime types are represented in this cluster. This cluster sees conflict levels similar to cluster 2, but with a much lower resilience index. Western, Central and Eastern Africa are well represented in this cluster.

Making data point sizes proportional to the refugee populations from each country in a given year allows us to visualize how the extracted dimensions are associated with human migration (Fig 2). The results suggest a significant association between Cluster 4, the high conflict cluster, and large refugee populations, supporting the idea of conflict as a main driver of human mobility. (It is important to note that the refugee data is used here for visualization purposes only and not part of the FAMD analysis). Some data points in Cluster 5, particularly those to the far right, also have significant refugee populations, while not as large as those in Cluster 4. The smallest refugee populations stem from observations in Cluster 1, where resilience and income are high.

### Conditions for conflict

**Fig 3:**
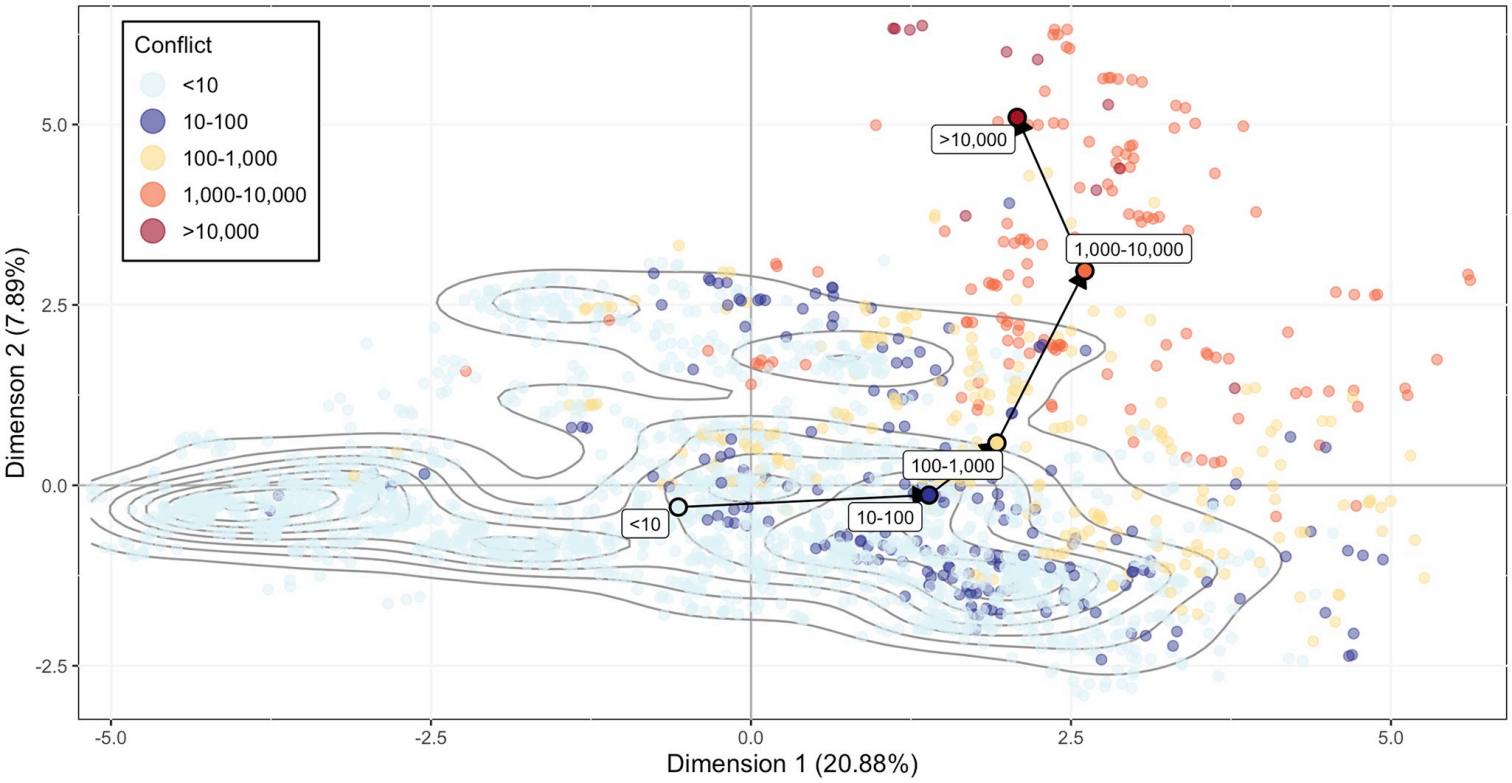
Conflict category mapped against the first and second dimensions. Each point represents an individual country-year observation, color-coded according to its level of conflict. Labeled category points correspond to the center of gravity of observations belonging to that category.

In the full dataset, conflict events are relatively rare, meaning there are far more observations with zero battle-related deaths than any other value. Observations with less than ten battle-related deaths are well dispersed across Fig 3. Higher conflict levels, however, are largely found on the right side of the map. Once a certain level of dimension 1 is crossed, conflict increases along dimension 2, shown by the upward turn of the conflict category progression. It is worth noting that this type of nonlinear/threshold behavior would have been undetected by multiple linear regression as done in some existing studies (e.g., Ref [4]), highlighting the importance of selecting appropriate methods for complex datasets.

While this method does not allow for statements on causality, these results suggest a threshold behavior with respect to conflict: high values of dimension 1–corresponding to low resilience, low control of corruption, low health, and high income inequality—are necessary, but not sufficient, for association with a high conflict level. Only with high values of dimension 2—a combination of high political terror, oil production, and water stress—high conflict level becomes a possibility. This can also be seen in the results of the hierarchical clustering analysis. Cluster 4, associated with the highest conflict levels, is found at the top right of Fig 2.

The resilience index, ND-GAIN, has a strong negative association with dimension 1, with observations with the highest resilience index concentrated on the left of Fig 2, corresponding to Cluster 1 (characterized by high income and a democratic regime). Middle and lower values of resilience index, however, are associated with more than one cluster each. Clusters 2 and 3 and Clusters 4 and 5 have very similar resilience index values, respectively. The ND-GAIN resilience index is made up of two values, a vulnerability score and a readiness score. Countries with high readiness and high vulnerability might have similar resilience index values to those with low readiness, but low vulnerability, giving way to more variability in observations with lower resilience index values. On the other hand, the only way to earn a very high resilience index value is to have both high readiness and low vulnerability, corresponding with highly concentrated Cluster 1. This further emphasizes the threshold behavior of conflict level: conflict is not the only threat to resilience and low resilience does not necessarily mean high conflict.

Our objective here was primarily methodological, reanalyzing an existing dataset to determine if new conclusions could be drawn with the application of more appropriate methods. This approach not only supports the use of factor and principal components analysis in developing clusters of nations more or less prone to conflict, relating variability in conflict to conditions such as resilience and regime type, but it also confirms and deepens findings from conflict research in multiple contexts. For example, in their critical path analysis of articles on conflict from 1983 to 2011, Van Holt *et al*. [9] found that research themes over time have evolved in their complexity. Conflict research initially focused on the role of resource availability in destabilizing democracy and leading to intrastate conflict, whether because resources are scarce and people are suffering or because valuable resources (like oil or other minerals) are plentiful but subject to looting or control concentrated in the hands of a few [22]. Later work applied theoretical insights from this earlier work to interstate and ethnic conflict and, more recently, conflict related to environmental and climate variables [23]. Our analysis here confirms that resource availability—reflected in GDP per capita (dimension 1), oil production, and water stress (dimension 2)—combined with the presence or absence of democracy (regime type, related to both dimensions), explains variations in conflict levels. In addition, our clustering analysis outlines combinations of variables associated with the presence or absence of different levels of conflict, thus deepening our understanding of conflict’s complexity.

### Country trajectories through time

**Fig 4:**
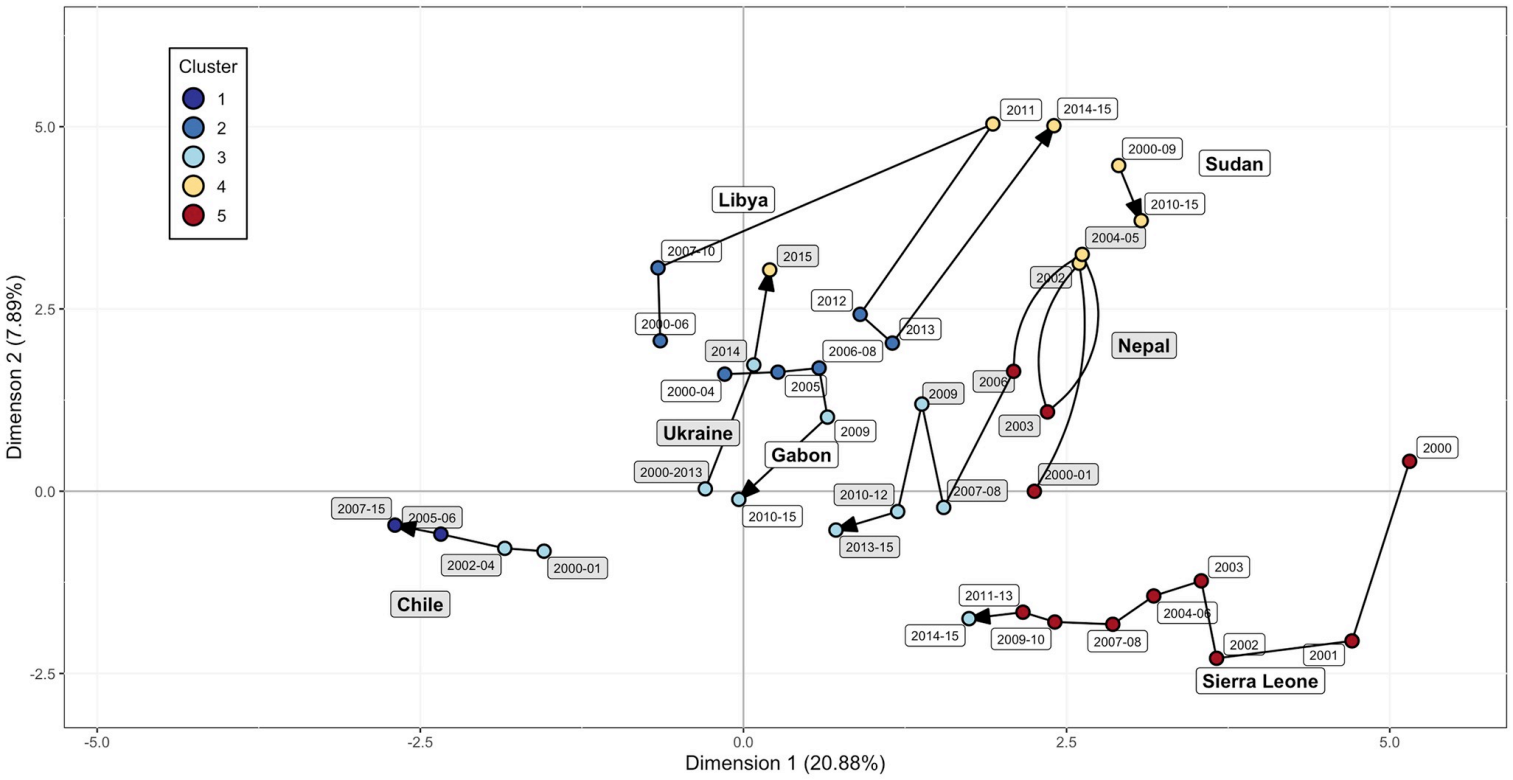
Seven countries and their movement through the first two dimensions in the years of the dataset. For points that are closely clumped together, their centers of gravity were used to ease readability. The interested reader is referred to S1 Table for the cluster memberships of all countries from 2000 to 2015.

Because observations represent one country in one year, this allows us to examine how a particular country moves in this two-dimensional space during the years covered by the dataset. Nepal provides one such example: a civil war in the country lasted from 1996–2006, with a ceasefire through most of 2003, a crucial time for negotiations between Maoist rebels and the established government [24]. This period of conflict saw high rates of “disappearances” and torture from government security forces [25]. Tensions rose again in 2009 due to conflict between the established government army and the newly elected Maoist government officials [26], accompanying a move from a ‘Partial Democracy’ to a ‘Partial Democracy with Factionalism’ regime type. These historical phases and events are reflected in Nepal’s trajectory in the map (Fig 4). Starting in 2000, Nepal was in Cluster 5, associated with lower income and low health indicators. As civil war conflict escalated, Nepal moved to Cluster 4, associated with high conflict and a high level of political terror. The ceasefire of 2003 brought Nepal back to Cluster 5 briefly before conflict resumed again. Following the conclusion of the war in 2006, and a rise in political tensions in 2009, Nepal settled into Cluster 3, associated with middle income and partial democracy, moving towards lower values of the first dimension. The path through conflict that Nepal took in this time period roughly follows the shape of the conflict curve seen in Fig 3.

Observations in Ukraine are fairly stable in Cluster 3 from 2000 through 2013. Ukraine enters Cluster 4 in 2015 after the onset of conflict in Crimea in 2014. Many narratives have been assigned to this conflict [27], offering independence, insurgency, and complex geopolitical relations as potential drivers of violence. For our analysis, Ukraine’s path is characterized by increases in dimension 2 in 2014 and 2015.

Libya moved directly from Cluster 2 to Cluster 4 with the onset of civil war in 2011, lasting eight months. The Arab Spring began in 2010 spreading across North Africa with Muammar Gaddafi being deposed in 2011. Following his death, there was a brief resolution of the conflict that brought it back to Cluster 2 before another civil war begins in 2014 reflecting the chaos following Gaddafi’s removal in which multiple groups were vying for power.

Civil war in Sierra Leone was declared over in 2002 after two years of declining conflict and international intervention. After that, increases in income and steady improvements to health indices like infant mortality and undernourishment moved the country towards Cluster 3.

The situation in Sudan represents a more stable conflict. Rather than violence peaking and falling, Sudan remained in Cluster 5 for the duration of the data time span. A 2005 peace agreement formally ended the nation’s civil war, but violent conflict and high political terror persisted [28]. A 2010 election moved Sudan from a ‘Full Autocracy’ to a ‘Partial Autocracy’ regime type, seen in the slight change in dimension 2 in Fig 4.

Gabon elected its first new president in over 41 years in 2009. This transition of power moved the country from a ‘Full Autocracy’ regime type to a ‘Partial Democracy with Factionalism’ regime type, without any significant conflict. This transition can be seen as observations jumped from Cluster 2 to Cluster 3 after 2009. In another example, elections in 2005 marked Chile’s movement from a ‘Partial’ to a ‘Full Democracy’. This, coupled with steadily increasing resilience index, characterizes its path from Cluster 2 into Cluster 1 (Fig 4).

## Conclusion

This study sought to answer the following research questions: What are the conditions associated with high levels of conflict? Is there any typology to illustrate these associations? Can we use this typology and visualization to understand the changes in a given country through time?

We found that conflict displayed threshold behavior with respect to the first two extracted dimensions. Generally, a country must first have a positive first dimension value (associated with low resilience index, low control of corruption, low income, and regime type), before conflict becomes a possibility. Then, increases in the second dimension (associated with oil production, political terror level, water stress, and regime type) determine the level of conflict. Future research should focus on what separates observations along the second dimension, bringing countries from conflict risk to actual conflict. Hierarchical cluster analysis was used to develop a typology of country-year characteristics—five clusters were identified and described. Several country trajectories through the two-dimensional space were then used to understand changes in characteristics through time. These are just a few examples of how this FAMD-based visualization can help us see a bigger picture and facilitate comparison across countries.

Along with the factors correlated with each dimension, the reduction in dimensionality of the phenomenon, capability to capture nonlinear behavior, and effective visualization, these findings have the potential to facilitate observation of regular patterns that can lead to better understanding of the emergence of conflicts. Future work should expand the datasets to include additional climate and environmental variables as well as investigate what separates observations along the second dimension, transitioning countries from situations of conflict risk to actual conflict. Our findings, particularly the variables associated with the first two dimensions, can help inform the selection of variables for use in broader multivariate linear and nonlinear models. They can also guide the application of more advanced causal inference techniques to uncover causal relationships between these drivers and conflict.

## Supporting information

S1 Table Cluster memberships of all countries from 2000 to 2015. (CSV)
